# Whole genome sequence-based analysis of *Staphylococcus aureus* isolated from bovine mastitis in Thuringia, Germany

**DOI:** 10.3389/fmicb.2023.1216850

**Published:** 2023-08-25

**Authors:** Amira A. Moawad, Hosny El-Adawy, Jörg Linde, Ines Jost, Gärtner Tanja, Hruschka Katja, Donat Karsten, Heinrich Neubauer, Stefan Monecke, Herbert Tomaso

**Affiliations:** ^1^Institute of Bacterial Infections and Zoonoses, Friedrich-Loeffler-Institut, Jena, Germany; ^2^Animal Health Research Institute, Agriculture Research Center (ARC), Giza, Egypt; ^3^Faculty of Veterinary Medicine, Kafrelsheikh University, Kafr El-Sheikh, Egypt; ^4^Tiergesundheitsdienst der Thueringer Tierseuchenkasse, Jena, Germany; ^5^Leibniz Institute of Photonic Technology (IPHT), Jena, Germany; ^6^InfectoGnostics Research Campus Jena e.V., Jena, Germany; ^7^Institute for Medical Microbiology and Virology, Dresden University Hospital, Dresden, Germany

**Keywords:** bovine mastitis, *Staphylococcus aureus*, MRSA, WGS, ST398, antimicrobial resistance, Thuringia

## Abstract

**Background:**

Bovine mastitis is a common disease of dairy cattle causing major economic losses due to reduced yield and poor quality of milk worldwide. The current investigation aimed to gain insight into the genetic diversity, antimicrobial resistance profiles and virulence associated factors of *Staphylococcus* (*S.*) *aureus* isolated from clinical bovine mastitis in dairy farms in Thuringia, Germany.

**Methods:**

Forty *Staphylococcus aureus* isolates collected from clinical bovine mastitis cases from 17 Thuringian dairy farms were phenotyped and genetically characterized using whole genome sequencing.

**Results:**

Out of 40 *S. aureus*, 30 (75%) were confirmed as methicillin resistant isolates. The isolates showed elevated antimicrobial resistance against penicillin, tetracycline and oxacillin, i.e., 77.5, 77.5, and 75%, respectively. Lower resistance rates were found against moxifloxacin, ciprofloxacin, gentamicin and trimethoprim/sulfamethoxazole, i.e., 35, 35, 30, and 22.5%, respectively. While resistance against clindamycin and erythromycin was rarely found (5 and 2.5%, respectively). All isolates were susceptible to linezolid, teicoplanin, vancomycin, tigecycline, fosfomycin, fusidic acid and rifampicin. These isolates were further allocated into five different sequence types: ST398 (*n* = 31), ST1074 (*n* = 4), ST504 (*n* = 3), ST582 (CC15) (*n* = 1) and ST479 (*n* = 1). These isolates were also assigned to seven clusters with up to 100 SNP which has facilitated geographical mapping and epidemiological distribution in Thuringia. Strains belonging to ST398 were classified into clusters 1, 2, 3, 4 and 7. The isolates of ST504 were of cluster 5, those of ST1074 were belonging to cluster 6. Resistance genes *bla*Z, *bla*I and *bla*R associated with penicillin resistance were found in 32 (80%) strains, all except one were belonging to ST398. Methicillin resistance associated *mec*A was identified in 30 (96.8%) isolates of ST398. All tetracycline and erythromycin resistant isolates were of ST398, and all harbored both *tet*M and *erm*A. About 90.3% of tetracycline resistant isolates assigned to ST398 were also carrying *tet*K gene. The point mutations *par*C_S80F, *gyr*A_S84L and *par*C_S80Y in *gyr*A and *par*C associated with quinolone resistance were found in all phenotypically resistant isolates to ciprofloxacin and moxifloxacin (*n* = 14). Sixty-eight virulence genes were identified among isolates. Both *luk*D/E and *lukM*/F-PV-P83 were identified in 22.5% of isolates, all were non-ST398.

**Conclusion:**

In this study, ST398 had the highest potential to cause disease and had a massive prevalence in bovine mastitis cases. Five different sequence types and seven clusters were identified in the federal state of Thuringia. The circulation of some clusters in the same region over several years shows the persistence of cluster-associated infection despite the intensive veterinary care. On the other hand, some regions had different clusters at the same year or in different consecutive years. Different sequence types and associated different clusters of *S. aureus* were geographically widely distributed among dairy farms in Thuringia. The findings of this study show that various clusters have the potential to spread over a large geographical scale. The detection of LA-MRSA on dairy farms, which is known for cabapility to widely spread among different groups of animals, humans and their environment urges for the implementation of national wide strategic programs. The identification of CA-MRSA among the isolates such as ST398 poses a significant risk for the transmission of such strains between animals and humans on dairy farms.

## Background

*Staphylococcus (S.) aureus* is among the most common pathogens associated with bovine clinical and subclinical mastitis in Germany and can cause difficult-to-treat cases (Tenhagen et al., 2018; Kadlec et al., 2019). *Staphylococcus aureus* infections primarily occur during lactation and rarely in the dry period causing increased somatic cell counts (SCC) in bulk tank milk (BTM) and severe losses of milk due to poor cure rates (Schnitt and Tenhagen, 2020).

Bovine mastitis is usually treated with β-lactams, fluoroquinolones, cephalosporins, macrolides, and aminoglycosides (Krömker and Leimbach, 2017; TÄHAV, 2018; Ziesch et al., 2018; Bolte et al., 2020; Preine et al., 2022). The first line therapy for udder intramammary infections is the administration of benzylpenicillin/cloxacillin. An alternative treatment is the intramammary (IMM) or intramuscular (IM) combination of cefalexin and kanamycin (Bolte et al., 2020). Additional systemic antimicrobial treatment depends on the veterinarian’s decision and is restricted to severe cases including methicillin resistant *S. aureus* (MRSA) infections.

Recent allelic variations of MRSA strains show the independent development and spread of these strains to the German federal states. Transmission between dairy farms within one federal state is more likely attributed to local races, trading networks and similar raising methods (Lienen et al., 2020). Additionally, MRSA can be transmitted between animals and farm personnel causing sporadic severe infections in humans such as otitis, wound infections, sepsis, dermatitis, pneumonia, and endocarditis (Goerge et al., 2017).

Methicillin-resistance is primarily encoded by *mec*A and/or its homolog *mec*C. These genes are part of the mobile genetic element called “staphylococcal cassette chromosome *mec*” (SCC*mec*) (Wendlandt et al., 2013; Schnitt and Tenhagen, 2020).

The cassette often harbors additional resistance genes against several classes of antibiotics including aminoglycosides, macrolides, fluoroquinolones and the last resort drugs such as linezolid. Therefore, MRSA are mostly considered ‘multi-drug resistant’ superbugs (Cuny et al., 2017). Bovine mastitis caused by *S. aureus* is also fortified by numerous virulence factors such as toxins, leukocidins, superantigens (SAs) such as enterotoxins *se*A–*se*Q, and toxic shock syndrome toxin 1 (*tsst*-1). The accessory genome apart from the core genome, comprises approximately 15%–25% of the total *S. aureus* genome, leading to great variations in genomic content. The accessory genome includes mobile genetic elements (MGEs), in which the virulence and antimicrobial resistance (AMR) associated genes are included (Alibayov et al., 2014; Feßler et al., 2018; Hoekstra et al., 2020).

Population genetic studies have shown that the genotypes of *S. aureus* are often correlated with host species-specificity. The bovine associated sequence types (STs) 97, 133, 151, 479, and 771 have been confirmed as the predominant sequence types incriminated in bovine mastitis in some previous studies (Schlotter et al., 2012; Hoekstra et al., 2020).

The livestock associated MRSA (LA-MRSA) are less likely to cause severe human infections due to a lack of Immune invasion cluster (IEC) genes, Panton-Valentine leucocidin (PVL) or genes encoding the toxic shock syndrome toxin (TSST) (Cuny et al., 2015). Nonetheless, LA-MRSA was identified in bovine mastitis in Germany with the likelihood of transmission to human (Kadlec et al., 2019; Lienen et al., 2020).

The LA-MRSA of clonal complex CC398 has a broad spectrum of hosts including humans and is increasingly reported as the predominant sequence type found in German dairy farms (Tenhagen et al., 2018; Lienen et al., 2020; Schnitt et al., 2020).

The aim of this study was to identify and compare the genotypes, antimicrobial resistance profiles, virulence factors and geographic mapping of clinical *S. aureus*/MRSA strains isolated from milk samples on dairy farms in Thuringia, Germany. Whole-genome sequencing (WGS) of strains and sequence data analysis were applied to assess and investigate the epidemiologic situation of clinical mastitis and to evaluate the arising potential of public health risk.

## Methods

### Identification and phenotypic characterization of *Staphylococcus aureus* isolates

Forty *S. aureus* isolates from milk were collected from cows with clinical mastitis from May 2020 to July 2022 from different 17 dairy herds in different locations in Thuringia (Supplementary Table S1). The samples were cultured and tested by the Animal Health Service of Thuringia (TGD) according to the guidelines of the German Veterinary Association (G.V.A, 2018) and international standards (NMC 2017) (Schnitt et al., 2020). All isolates were further investigated at the Institute of Bacterial Infections and Zoonoses, Friedrich-Loeffler-Institut, Germany. In order to identify and differentiate MRSA, all isolates were cultivated on Chromogenic agar medium (Mast Diagnostica GmbH, Reinfeld, Germany) at 37°C for 24 h under aerobic conditions. All isolates were confirmed as *S. aureus* using MALDI-TOF MS (Manukumar and Umesha, 2017; Yu et al., 2022). The interpretation of MALDI-TOF MS results was performed according to the manufacturer’s recommendation. The reference database was provided by Bruker (MBT-BDAL-8468). Multiplex real-time PCR assays were also performed for genus and species identification (Velasco et al., 2014; Wang et al., 2014).

### Phenotypic antimicrobial susceptibility testing

The minimum inhibitory concentration (MIC) testing for *S. aureus* isolates was performed using a VITEK-2 system (VITEK-2, bioMérieux Deutschland GmbH, Nürtingen, Germany) with VITEK 2 AST-P592 Group B *Staphylococci*, *Enterococci*, and *Streptococci* Susceptibility Testing Cards (bioMérieux Deutschland GmbH, Nürtingen, Germany) according to the manufacturer’s instructions. The tested antimicrobial agents included in the VITEK 2 AST cards were penicillin, ampicillin, oxacillin, imipenem, gentamicin, ciprofloxacin, moxifloxacin, erythromycin, clindamycin, linezolid, teicoplanin, vancomycin, tetracycline, tigecycline, fosfomycin, fusidic acid, rifampicin, and trimethoprim/sulfamethoxazole. The interpretation of results was carried out according to the EUCAST standard (Version 13.0, 2023/NAK Deutschland).

Phenotypic methicillin/oxacillin resistance was also confirmed using E-TEST oxacillin (0.016–256 μg/mL) gradient strips (bioMérieux, Baden-Württemberg, Germany) (CLSI, 2020). For quality control of susceptibility testing, the *S. aureus* (DSM 2569) and *E. coli* (ATTC^®^ 352,189) reference strains were used (CLSI, 2018).

### DNA extraction and purification

A loopful from each fresh culture was suspended in 1 mL sterilized phosphate buffer saline (PBS) and heat inactivated at 96°C for 30 min. Genomic DNA was extracted and purified from bacterial cultures using QIAGEN Genomic-tip 20/G Kit (Qiagen GmbH, Hilden, Germany) according to the instructions of the manufacturer with a prior in-house modification step, i.e., adding of a lysis mixture (10–20 μL lysostaphin, 20 μL lysozyme, 2 μL ribonuclease A (2 μL of 10 mg/mL) and 45 μL proteinase K) followed by an incubation at 37°C for 2 h with slight shaking.

The concentration and quality of eluted DNA was determined photometrically using a Colibri spectrophotometer (Titertek, Berthold Technologies GmbH & Co. KG, Germany) and additionally measured using a Qubit 3 fluorometer (Fisher Scientific GmbH, Dreieich, Germany). The prepared DNA was preserved at −20°C for further investigations.

### Determination of the presence of *mec*A

The PCR was carried out in a 25 μL reaction mix including 10 μL of LC480 Probes Master PCR Reaction Mix (Roche Diagnostics, Mannheim, Germany), 3.25 μL of PCR water (Roche Diagnostics, Mannheim, Germany), 1 μL of each primer, 0.25 μL of each probe, 5 μL of template and H_2_O for the non-template control. Amplification reactions were performed in a CFX96 Touch Real-Time PCR Detection Thermocycler System (Bio-Rad Laboratories GmbH, Feldkirchen, Germany).

PCR conditions were as follows; 1 cycle (50°C, 2 min.), 1 cycle (95°C, 10 min.) and 50 cycles (95°C, 20 s; 60°C, 40 s) (Velasco et al., 2014; Wang et al., 2014). *S. aureus* (DSM 2569) was used as positive control in each reaction.

### Whole genome sequencing

The extracted DNA was sequenced using an Illumina MiSeq2000 platform. Sequencing libraries were created using the Nextera XT DNA Library Preparation Kit (Illumina Inc., San Diego, CA). Paired end sequencing producing 300 bp long reads was performed on an Illumina MiSeq instrument according to the manufacturer’s instructions (Illumina Inc., San Diego, CA). Raw sequencing data were deposited at the European Nucleotide Archive (ENA) as BioProject PRJEB61659. The bioinformatic analysis started with quality control of the raw paired end reads. The Linux based bioinformatics pipeline WGSBAC v. 2.2.0^1^ was used for data analysis. Unless other settings are mentioned, all tools were used in their default standard settings. For quality control of raw reads, FastQC v. 0.11.7 (Andrews, 2018) was used and the coverage was calculated. Based on raw reads, WGSBAC performed assembly using Shovill v. 1.0.4.^2^ The quality of the assembled genomes was checked viaQUAST v. 5.0.2 (Bankevich et al., 2012). In order to check for potential contamination on both reads and assemblies, the pipeline uses Kraken 2 v. 1.1 (Wood et al., 2019) with the database Kraken2DB. For the detection of genetic markers for antimicrobial resistance and virulence, WGSBAC uses the software ABRicate (v. 0.8.10)^3^ and the databases ResFinder (Florensa et al., 2022), NCBI (Sayers et al., 2020), and Virulence Factor Database (VFDB) (Chen et al., 2005). Moreover, WGSBAC used AMRFinderPlus (v. 3.6.10) (Feldgarden et al., 2019) which detects genes and point mutations leading to AMR. AMRFinderPlus is used in organism-specific settings (i.e., *Staphylococcus aureus*). Platon is used for detection of plasmid borne contigs (Schwengers et al., 2020).

For genotyping, WGSBAC uses classical multilocus sequence typing (MLST) on assembled genomes using the software mlst v. 2.16.1 that incorporates the PubMLST database for the seven housekeeping genes of *S. aureus.*^4^ For high resolution genotyping, WGSBAC performed mapping-based genotyping using core genome single nucleotide polymorphisms (cgSNPs) identified by Snippy v. 4.3.6.^5^ The genome of *S. aureus* NCTC 8325 (accession NC_007795.1) was used as reference. RAxML (Randomized Axelerated Maximum Likelihood) v. 8 (Stamatakis, 2014) was used for phylogenetic tree construction based on cgSNP analysis. The tree was rooted to the reference genome and visualized using the interactive Tree of Life (iTOL) v. 4 web tool.^6^ Hierarchical clustering within the statistical language R was performed based on the pairwise SNP distances.

## Results

All 40 isolates were identified as *S. aureus* using MALDI-TOF MS and confirmed by real-time PCR (Supplementary Table S1). Out of 40 *S. aureus*, 30 (77.5%) were confirmed as methicillin resistant *S. aureus* (MRSA) isolates by growing on chromogenic agar media and amplification of the *mec*A gene by real-time PCR (Supplementary Table S1).

1. **Phenotypic antimicrobial resistance profiles**

Thirty (75%), 31 (77.5%) and 31 (77.5%) *S. aureus* were phenotypically resistant to oxacillin, penicillin and tetracycline, respectively. Antimicrobial resistance against moxifloxacin, ciprofloxacin, gentamicin and trimethoprim/sulfamethoxazole was detected in 14 (35%), 14 (35%), 12 (30%) and 9 (22.5%) strains, respectively. Few isolates were resistant against clindamycin (2/40, 5%) and erythromycin (1/40, 2.5%). All isolates were sensitive to linezolid, teicoplanin, vancomycin, tigecycline, fosfomycin, fusidic acid and rifampicin. All oxacillin-resistant isolates were also resistant to penicillin and tetracycline (Table 1). Twenty-four strains (60%) were resistant to at least three classes of antibiotics and confirmed as multi-drug resistant (MDR) (Supplementary Table S1).

**Table 1:** Phenotypic resistance profiles of 40 *S. aureus* isolated from clinical bovine mastitis in Thuringia, Germany.

Antimicrobial class	Antimicrobial agent	Resistance	
		*n*.	%
Beta-lactam antibiotics	Penicillin	31	77.5
	Oxacillin	30	75
Aminoglycosides	Gentamicin	12	30
Fluoroquinolones	Ciprofloxacin	14	35
	Moxifloxacin	14	35
Macrolides	Erythromycin	1	2.5
Lincomycin	Clindamycin	2	5
Oxazolidinones	Linezolid	0	0
Glycopeptides	Teicoplanin	0	0
	Vancomycin	0	0
Tetracyclines	Tetracycline	31	77.5
	Tigecycline	0	0
Phosphonic antibiotics	Fosfomycin	0	0
Fusidane class	Fusidic acid	0	0
	Rifampicin	0	0
Sulfonamides plus trimethoprim	Trimethoprim/Sulfamethoxazole	9	22.5

2. **Genetic characterization of *Staphylococcus aureus* isolates**

Five different sequence types (ST) were identified. The majority of isolates (31/40) were assigned to ST398. Of note, chimerism with CC9, as frequently observed in livestock ST398 (Fetsch et al., 2017), was ruled out, based on an absence of Q2G1R6-*cstB* as well as on a presence of C5Q1F1-*ycjY* and G7ZTC1 (Monecke et al., 2018).

**Figure 1:**
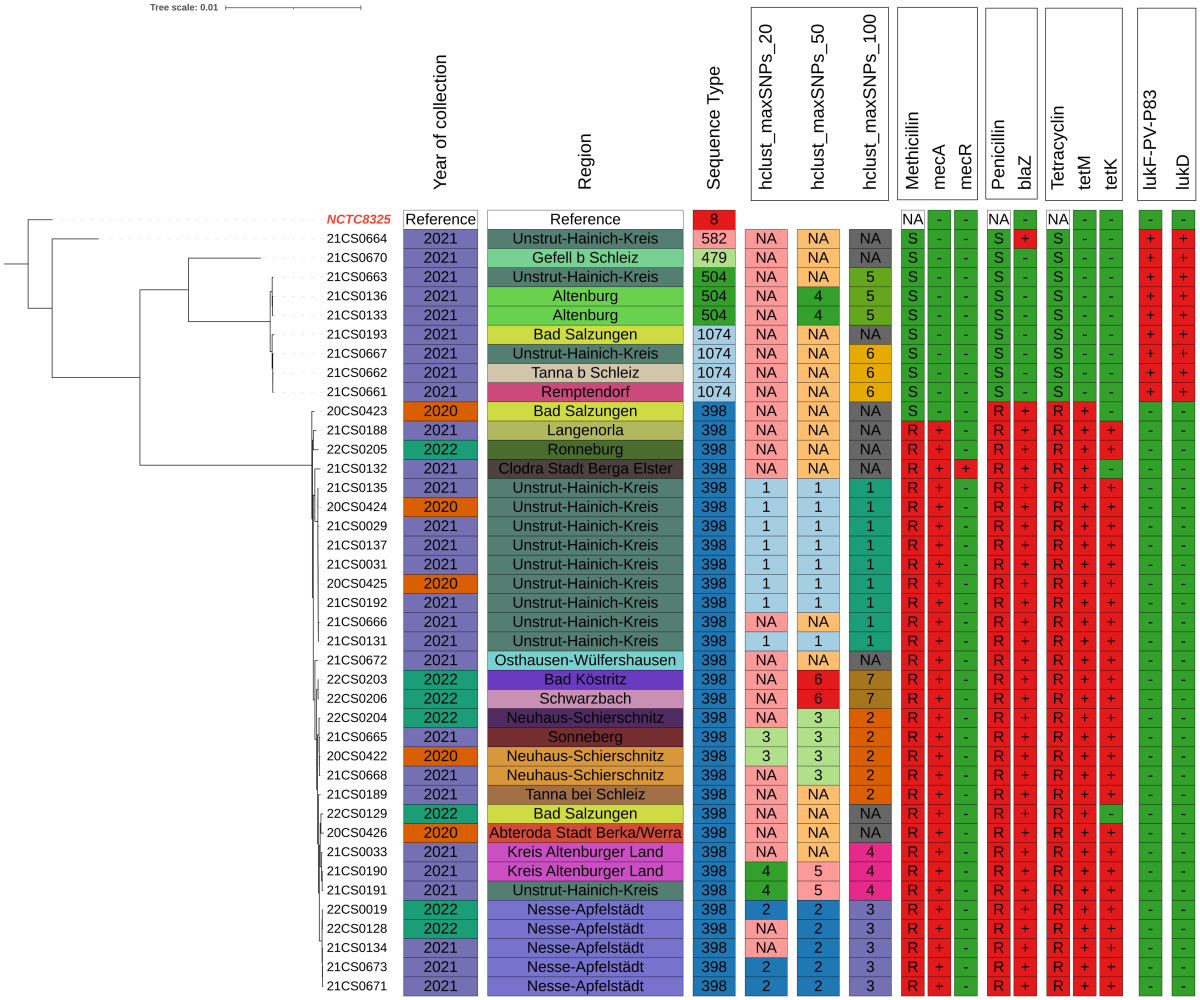
Phylogenetic analysis tree of 40 *Staphylococcus aureus* isolated from clinical mastitis cases from dairy farms in Thuringia using WGS analysis. The tree was rooted to the reference genome and visualized using the interactive Tree of Life (iTOL) v. 4 web tool (https://itol.embl.de/login.cgi). Hierarchical clustering within the statistical language R was performed based on the pairwise SNP distances. Data included are; Survey year, ST types, city, hclust maxSNPs_20, 50, and 100. Phenotypic resistance to penicillin, methicillin and tetracycline. Resistance genes; *mec*A/C, *bla*Z, *tet*M and *tet*K. Virulence genes *luk*D, E and *luk*F-PV.

**Figure 2:**
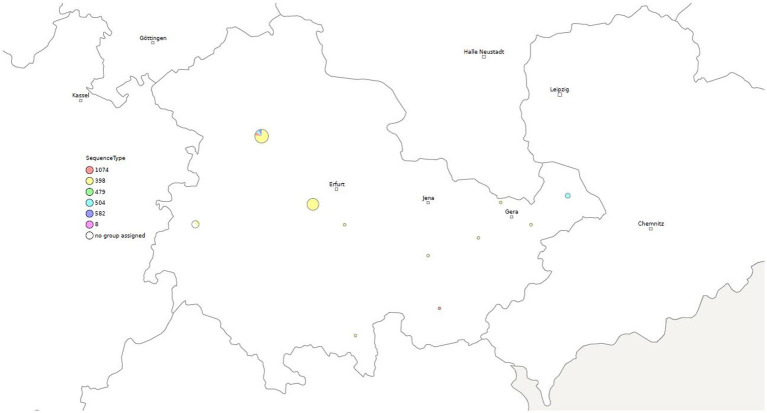
Geographical distribution of different sequence types (STs) of *S. aureus* among clinical mastitis cases from dairy farms in Thuringia.

Four isolates were identified as ST1074 and three isolates as ST504. Both of these two sequence types belong to CC705. One isolate each was assigned to ST479 and ST582 (CC15) (Supplementary Table S1 and Figures 1, 2).

3. **Genetic characterization of antimicrobial resistance**

Thirty isolates were *mec*A positive, fully in accordance to phenotypic test results. All MRSA isolates identified in this study belonged to ST398. The most abundant SCC*me*c element was the SCC [*me*c VT + *czrC*] composite element as previously sequenced in SO385; GenBank AM990992.1. This was identified in twenty-six isolates. Three isolates harbored other variants of SCC*mec* VT. In one of them, an additional *ccrA*-1 recombinase gene was detected. Finally, a single isolate carried a SCC*mec* IVa element.

The penicillin resistance causative gene *bla*Z was was identified in 32 (80%) of isolates. All except one belonged to ST398 (one isolate was on ST582/CC15).

A simultaneous presence of both tetracycline resistance genes, *tet*M and *tet*K, was identified in 28 isolates that were all belonging to ST398, while *tet*M alone was identified in all ST398 (Chen et al., 2005) isolates. Data for all other resistance associated genes are shown in Supplementary Table S1.

4. **Relatedness between phenotypic and genotypic resistance profiles**

The isolates showed high similarity in phenotypic and genotypic resistance profiles for most of the tested drugs (Figure 1 and Supplementary Table S1).

The resistance gene *bla*Z gene was identified in all isolates that were phenotypically resistant to penicillin (100%). All phenotypically methicillin resistant isolates harbored *mec*A (100%). All tetracycline resistant isolates carried either *tet*M alone or both, *tet*K and *tet*M. All trimethoprim/sulfamethoxazole and erythromycin resistant isolates carried either *dfr*G or *dfr*K and *erm*A, respectively.

Additionally, a concordance of 100% was observed in case of ciprofloxacin and moxifloxacin resistance in relation to the carriage of point mutations related to the drug resistance.

5. **Geographical distribution of genetic variants**

In order to study the phylogeographic variation of *S. aureus* in Thuringia, clustering of cgSNP general genomic clusters (100 cgSNPs) and more closely related strains (20 cgSNP) were applied. Thirty out of 40 strains (75%) were clustered into seven clusters. The most prominent clusters were; 1 (22.5%), 2 (12.5%) and 3 (12.5%). The distribution of all clusters is shown in Figure 1 and Supplementary Table S1.

Clustering based on 100 cgSNPs has higher discriminatory power than MLST. Therefore, strains belonging to ST398 were separated into clusters 1, 2, 3, 4 and 7. The isolates of cluster 5 were identified as ST504. Isolates of cluster 6 were belonged to ST1074. The predominant sequence type (ST398) was confirmed in all MRSA of 5 different clusters and circulated in 14 locations (Figures 1, 2).

To further refine clustering of the closely related strains, 20cgSNPs were used as a cutoff. This clustered 15 out of 40 strains (37.5%) into four clusters. Cluster 1 contained eight strains which were all isolated in the district Unstrut-Hainich-Kreis between 2020 and 2021. Three strains all isolated in the district Nesse-Apfelstädt between 2021 and 2022 were grouped within cluster 2. Cluster 3 consisted of two strains collected in Neuhaus-Schierschnitz and Sonneberg in 2020 and 2021. Within cluster 4 two strains each from districts Altenburger Land and Unstrut-Hainich-Kreis in 2021 were grouped.

6. **Geographical distribution of antimicrobial resistance**

The geographical distribution of phenotypic antimicrobial resistance of *S. aureus* isolates based on 100 cgSNPs is illustrated in Figure 1 and Supplementary Table S1. Interestingly, all *S. aureus* of cluster 1 isolated in 2020 and 2021 in Unstrut-Hainich-Kreis had the same phenotypic antimicrobial resistance profiles. The same effect was noticed for isolates of clusters 3, 4, 6, and 7, that were isolated in 2020 and 2021 from different locations (Supplementary Table S1).

On the contrary, isolates of clusters 2 and 5 did not share the same phenotypic resistance profile among the same cluster.

7. **Virulence associated genes**

Sixty-eight virulence genes were identified in 40 *S. aureus* isolates using WGS (Supplementary Table S1). They were assigned to five functional categories (adherence related virulence factors (*n* = 14), toxins; including toxin system genes (*n* = 17), enterotoxins and enterotoxin-like genes (*n* = 9), iron acquisition and metabolism-related genes (*n* = 7), host immune evasion genes (*n* = 8) and exoenzymes (*n* = 13)).

Regarding leukotoxins, the *hlg*-locus comprising of *luk*S, *luk*F and *hlg*A were present in all isolates, as they were the chromosomal genes encoding a bi-component leukocidin *luk*A/B (=*luk*G/H). The genomic island associated leukotoxins *luk*D/E were present in nine out of forty isolates, i.e., all CC15, CC479 and CC705 isolates, but absent from all CC398. The phage-borne leukocidin genes *luk*M/*luk*F-P83 were identified in the CC479 and CC705 sequences, i.e., in 9 out of 40 isolates. The genes encoding Panton-Valentine leukocidin, *luk*F/S-PV were absent from all sequences.

The enterotoxin gene cluster *egc*, comprising *seg*, *sei*, *selm*, *seln*, *selo* and *selu* was detected in the CC479 and CC705 sequences, i.e., in 8 out of 40 isolates. The CC705 isolates additionally harbored an enterotoxin gene homologue, ORF CM14. Only a single isolate (the CC398 MRSA with the composite SCC*mec* VT + *ccrA1* element) isolate carried one of the “classical” enterotoxin genes, *sea*. This sequence also contained genes *sak*, *chp* and *scn* (encoding staphylokinase, chemotaxis-inhibiting protein CHIPS and a staphylococcal complement inhibitor) that are frequently co-localized with *sea* on hemolysin-beta-converting phages in *S. aureus* strains from humans.

Genes encoding epidermal cell differentiation inhibitors or exfoliative toxins were not identified in any genome sequence examined.

## Discussion

*Staphylococcus (S.) aureus* is one of the most prevalent bacterial species incriminated in clinical and subclinical bovine mastitis infections as reported by different studies that have focused on *S. aureus* in dairy herds (Tenhagen et al., 2006; Minst et al., 2012; Tenhagen et al., 2014; Bolte et al., 2020). The pathogen has been isolated very often from bovine mastitis outbreaks in Germany and has developed multiple resistances (Tenhagen et al., 2006; Minst et al., 2012; Bundestierärztekammer, 2015; Zimmermann, 2017; Bolte et al., 2020).

In this study, we examined the presence of both methicillin resistant and methicillin sensitive *S. aureus* (MRSA/MSSA) associated with clinical mastitis in dairy cattle in addition to associated antimicrobial resistance and virulence factors in 40 isolates from Thuringian dairy farms.

The most conspicuous result was the high prevalence of MRSA. Previous studies from the same region indicated an MRSA prevalence of a mere 2% among bovine mastitis isolates collected 10 years ago in the same geographical region (Schlotter et al., 2012). Although different sampling procedures and low isolate number hinders a substantiated epidemiological assessment, it is assumed that CC398 MRSA among cattle mastitis cases is not that rare anymore.

The MLST (generated using WGS data) in this study has clustered *S. aureus* isolates into sequence types (STs). The majority of isolates were assigned to ST398 (77.5%), ST1074 (10%), ST504 (7.5%), ST582 (2.5%) and ST479 (2.5%). ST504 and ST1074 are related to each other and both belong to CC705, which is a well-known bovine lineage. ST582 belongs to CC15 which is common among humans, suggesting anthropo-zoonotic transmission. The detection of these CC479, CC705 and CC398 strains from bovine milk is not surprising as it is consistent with most studies nationally and internationally (Hoekstra et al., 2018, 2020; Lienen et al., 2020; Schnitt et al., 2020; Campos et al., 2022). Except in Asia, where ST9 is more prevalent (Annamanedi et al., 2021), ST398 is the most common LA-MRSA lineage worldwide, which is responsible for a wide range of different infections, from minor skin lesions to invasive infections and even fatalities in humans. However, the presence of distinct strains within CC398 in this study should be noted, out of which some are adapted to humans and carry IEC genes, sometimes even PVL while others are associated only with livestock (Kashif et al., 2019).

Previous molecular examination of many LA-MRSA ST398 strains confirmed the low pathogenicity and low number of associated virulence factors in these strains (Kraushaar et al., 2017; Ji et al., 2021). These findings are in accordance with the low number of virulence factors identified in ST398 in the current study.

According to the national reference center for staphylococci in Germany, about 3.7% of human clinical MRSA infections are associated with LA-MRSA ST398 (Schnitt and Tenhagen, 2020). The transmission of LA-MRSA between humans and animals, including dairy cows has only been reported on a few occasions (Cuny et al., 2015; Schnitt and Tenhagen, 2020).

Many mastitis outbreaks caused by LA-MRSA ST398 have been reported in Germany (Tenhagen et al., 2018; Hansen et al., 2019). These findings agree with our finding that ST398 is the predominant identified ST in 77.5% of our isolates.

In the current study, we also found only one (2.5%) isolate assigned to ST479 which is known for its dominant lineage associated with bovine mastitis that carries variable resistance and virulence genes in European countries, including Germany (Hoekstra et al., 2020).

Moreover, health care acquired methicillin resistant *S. aureus* (HA-MRSA) was found in dairy cows in Germany (Tenhagen et al., 2018) and indeed HA-MRSA ST582 was identified once (2.5%) among isolates in this study.

### Antimicrobial resistance

Antimicrobial resistance (AMR) usually spreads among *S. aureus* isolates due to the selective pressure as a result of extensive use of antimicrobials to treat bovine mastitis. The European Network for Optimization of Veterinary Antimicrobial Treatment (ENOVAT), COST Action CA 18217, has listed bovine mastitis as a priority infectious disease that contributes significantly to antimicrobial consumption in animals in European countries (Preine et al., 2022).

In the current study, resistance to the antibiotic classes, i.e., aminoglycosides, β-lactams, trimethoprim-, tetracyclines, macrolides, streptogramin, lincosamides, quinolones and phosphonic antibiotics was confirmed showing notable differences between the dairy farms of the current study.

The phenotypic resistance was mostly in agreement with bioinformatic predictions made from sequence data mined for AMR genes. Resistance to oxacillin was mediated in all MRSA strains in this study by the *mec*A gene while the variant *mec*C gene was not detected in this study. This gene is usually restricted to a few clonal complexes, including CC130, CC599 and CC425. The former ones occur in small wild mammals such as hedgehogs, also locally in Thuringia (Monecke et al., 2016). The latter has also been found in cattle (García-Álvarez et al., 2011), but CC425-MRSA-XI have, to the best of our knowledge, not yet been identified in Thuringia.

All oxacillin resistant isolates in this study were also resistant to penicillin and tetracycline. This finding might be attributed to the fact that hydrophilic molecules such as β-lactams, tetracyclines and some fluoroquinolones are particularly affected by the same changes in permeability of the outer membrane as a mechanism of bacterial resistance (Munita and Arias, 2016). It is a fact that all CC398 MRSA carry at least one *tet* gene, and usually even several.

The blanket dry cow therapy (DCT) with long acting β-lactam antimicrobials, including penicillin and cloxacillin, is still often used to treat intramammary *S. aureus* infections. Hence, it could be considered as a factor that might pose a selective pressure favoring the dissemination of MRSA strains (Schnitt et al., 2020).

Antimicrobial resistance against penicillin is primarily attributed to enzymatic inactivation of β-lactams and target site modification. Not unexpectedly, phenotypic resistance to penicillin was identified in 77.5% of all *S. aureus* isolates and in 100% of MRSA isolates in this study. This resistance was confirmed by identification of β-lactamase encoding genes *bla*I, *bla*Z, *bla*R and *bla*PC. Tenhagen et al., have accordingly earlier reported a 100% penicillin resistance in MRSA isolates in German dairy herds (Tenhagen et al., 2018). Another study recorded that *bla*Z was found in 87.9% of MRSA isolates from dairy farms (Lienen et al., 2020).

Tetracyclines are extensively used in veterinary medicine, thus promoting the occurrence of tetracycline resistant strains. Tetracycline resistance is often mediated by *tet*L, *tet*K and *tet*M, which code for membrane associated efflux proteins of the Major Facilitator Superfamily. In this study, phenotypic resistance to tetracycline was confirmed in 77.5% of all isolates, which was mediated by *tet*K and/or *tet*M. These findings were in agreement with results of other studies where all MRSA strains from dairy farms were resistant to tetracycline (Fessler et al., 2012; Kadlec et al., 2019; Lienen et al., 2020). Besides, tetracycline resistance was detected in 95.1% of the MRSA in BTM from German dairy farms (Tenhagen et al., 2018).

Macrolide, lincosamide, and streptogramin resistance in staphylococci is based on the presence of one or more *erm* genes that modify the target site(s) in the 23S rRNA and thereby inhibit the binding of these drugs. Resistance to erythromycin was rare among strains examined (2.5%) in this study. This finding fits well with reports that macrolide resistance in *S. aureus* retrieved from bovine mastitis is low (Lienen et al., 2020). The *erm*A gene, known to confer resistance to macrolides, lincosamides and streptogramin B (Leclercq, 2002), was identified in the sole erythromycin resistant isolate in this study. The isolate was also resistant to clindamycin.

In contrast to most mastitis therapeutics, which are administered by direct injection into the infected mammary gland, fluoroquinolones are usually administrated systemically in several Scandinavian countries. These countries have used a common strategy for treating clinical mastitis since several years. According to the “Nordic Guidelines for Mastitis Therapy” a restrictive use of penicillin is followed by a reduced application of cephalosporins and quinolones. Both cephalosporins and quinolones are considered as critically important antimicrobials (CIAs) belonging to human reserved list (Landin et al., 2011; Preine et al., 2022). Fluoroquinolones are used in dairy cattle and calves in Germany. However, published data on use and resistance against these drugs are limited. In this study, phenotypic resistance to quinolones, i.e., ciprofloxacin and moxifloxacin was found in 35% of all isolates. These isolates were all identified as MRSA. The resistance was reflected by the presence of point mutations *par*C_S80F, *gyr*A_S84L and *par*C_S80Y in all quinolone resistant isolates. Ciprofloxacin resistance in *S. aureus* is mediated by point mutations that alter ciprofloxacin targets, including topoisomerase IV (*grl*A, *grl*B) and DNA gyrase (*gyr*A, *gyr*B). The most common single point mutations, such as *grl*A E84K and *grl*A S80F/Y, are often found in ciprofloxacin resistant clinical isolates (Papkou et al., 2020). Amongst MRSA isolates from German dairy herds, quinolone resistance was either not found (Spohr et al., 2011; Kadlec et al., 2019), or at a low prevalence (8.3%) (Kreausukon, 2011).

Resistance to trimethoprim was detected in 22.5% of all isolates and 30% of MRSA isolates in the current study. That was quite lower than results obtained in previous studies in 2012 and 2021, where 43.2% or more than half of MRSA isolates from dairy farms were resistant to trimethoprim (Fessler et al., 2012; Lienen et al., 2020). The resistance to trimethoprim in this study was mediated by the *dfr*G and/or *dfr*K genes that were identified in all phenotypically resistant isolates. Both genes were only identified in CC398 isolates, which was in agreement with other studies that identified *dfr*K only in CC398-MRSA isolates in German dairy herds (Fessler et al., 2010; Lienen et al., 2020).

Staphylococci are non-target bacteria to pleuromutilin. However, the use of these drugs (instead of their use) in pig farming selects for multi resistant MRSA (Lienen et al., 2020). In this study, pleuromutilin resistance was confirmed by the detection of *vga*A or *vga*E genes. Thus, a hypothesis of pig-transmission should be taken in consideration. The *vga*A gene was reported in MRSA strains from several farms located in different German federal states (Schwendener and Perreten, 2011; Feßler et al., 2018). Additionally, Hauschild et al, detected the *vga*E gene in CC398 in dairy cattle (Hauschild et al., 2012). Although the *vga*A gene was reported as the most widespread among the *vga* genes (Feßler et al., 2018), in the current study the *vga*E (12.5%) gene was more common than *vga*A (2.5%) across the MRSA strains.

Aminoglycosides are widely used in veterinary medicine (EMA, 2017). Enzymatic inactivation (acetylation and phosphorylation) are the main mechanisms for the resistance against them. Phenotypic resistance against gentamicin was identified in 36.7% of MRSA isolates in this study. All gentamicin resistant isolates belonged to ST398 MRSA strains and carried the resistance associated genes *ant*(6)-Ia, *aac*(6′)-Ie-aph(2″), *aad*D1, *ant*(9)-Ia, *ant*(4′)-Ia and aph(2″)-Ih. In another study, the resistance against gentamicin among MRSA CC398 isolated from German dairy farms was very similar to our results (38.4%) (Fessler et al., 2012).

An earlier study by Schlotter et al. in German dairy herds recorded phenotypic resistance to both linezolid and teicoplanin (38.9 and 5.6%) in MRSA-CC130 isolates, while resistance to vancomycin, tigecycline, fosfomycin, or rifampicin was not observed amongst the isolates (Schlotter et al., 2014). In this study, none of the isolates showed resistance to linezolid, tigecycline, vancomycin, fosfomycin or teicoplanin.

The presence of MDR efflux pumps *nor*A, *Lmr*S, *mep*A and *mep*R genes was observed in all *S. aureus* isolates in this study. However, not all of them were associated with expressed phenotypic drug resistance. Of note, other studies reported the high prevalence of the same elements in *Staphylococcus* spp. (Antiabong et al., 2017).

The data of earlier studies could be confirmed in the current study. Lower values of resistance against various agents might be caused by the limited number of strains investigated. There are obvious indications in this study for transmission of *S. aureus* strains from carrier host species like human or pig to cattle.

### Virulence factors

Virulence factors (VFs) such as enterotoxins, leukocidins and haemolysins produced by staphylococci isolated from milk, could generate potential public health implications for consumers. *S. aureus* has the ability to survive inside mammary epithelial cells and immune cells after invading the teat canal (Mullarky et al., 2001; Barkema et al., 2006). Therefore, infections may persist over a long period and antimicrobial therapy is often hindered.

The distribution of a large number of VFs in isolates of this study was determined. These VFs were grouped into five functional categories.

Haemolysins α, β, γ and δ toxins of *S. aureus* play a major role in its pathogenicity. All 4 hemolysin genes were identified in all *S. aureus* isolates, which is consistent with other reports (Schmidt et al., 2017).

Leukocidins include Panton Valentine leukocidin (*luk*F/S-PV, lukM/lukFP83), *luk*A/B and *luk*D/E. They cause structural changes that facilitate membrane insertion and formation of octameric pores. In the current study, *luk*D/E and *lukM*/F-PV-P83 were found in non-ST398 isolates.

Enterotoxins are heat stable and usually persist after heat treatment in dairy products and remain active in the gastrointestinal system after ingestion. Enterotoxins are associated with staphylococcal food poisoning caused by cow milk and other dairy products (Benkerroum, 2018). *Sea*, *seb*, *sec*, *sed* and *see* represent the “classical types” of enterotoxins.

The distribution of enterotoxin genes here differed greatly among isolates and different STs. While among the most prevalent ST (398), only a single isolate contained the *sea* gene (*n* = 1), other enterotoxins *sem*, *sei*, *seu*, and *sen* were identified in other STs.

## Conclusion

In the current study, *S. aurus* isolated from bovine clinical mastitis cases from farms of a single German federal state were investigated. Considering the limited number of isolates investigated in this study, a significant number of different sequence types and associated clusters was found and geographically widely distributed among dairy farms. Of note, the prevalence of MRSA was much higher than in previous studies on bovine mastitis in the same region. Persistance of clusters in the same region for different years shows that veterinary control measures do not have the impact to control disease. Detection of LA-MRSA in dairy farms, which is known for cabapility to spread easily among different groups of animals, humans, and in the environment suggests the need for urgent implementation of national wide strategic programs as already demanded in the animal health law (AHL) 2016 and recently confirmed by various EFSA reports. The identification of CA-MRSA such as ST398 among isolates poses a high risk for transmission and interplay of such strains between animals and humans on the dairy farms. The hygienic surveillance and awareness among the workers and veterinarians should be intensified in the future in order to expand the AMR and safeguard public health integrity.

The findings of this study show that various clusters have the potential to spread over a large geographical area (e.g., cluster 4), where some other clusters were more geographically restricted (e.g., cluster 1 and 3).

The mandatory antimicrobial susceptibility testing proves to be crucial in combination with prudent use of antimicrobials to avoid the emergence of resistance. The proposed action favours three of the current agricultural regulations, i.e., implementation of EU legislation, increase of income through avoiding losses due to reduced milk yield and decrease the veterinary costs. Most importantly is increasing the animal welfare through reducing suffering from clinical and subclinical mastitis infections in dairy farms in Germany.

As bovine mastitis caused by *S. aureus* especially MRSA is a world-wide problem, we aimed in this study to spot light on the situation in Thuringia as one of German federal states in Germany having relatively high number of cattle farms for milk production and suffering from recurrent mastitis problems of different causes.

This study aimed also to update the situation of the bovine mastitis caused by *S. aureus*/MRSA and using WGS for full genetic characterization in addition to antibiotic resistance prediction. These are among the main objectives of a joint project to innovate a fast robust tool for rapid detection of AMR in field cases of mastitis.

## Data availability statement

The datasets presented in this study can be found in online repositories. The names of the repository/repositories and accession number(s) can be found in the article/Supplementary material.

## Author contributions

AAM, HE-A, HT, and HN: conceptualization. AAM, JL, HT, HE-A, IJ, GT, HK, and DK: methodology. JL and SM: software and validation. AAM, JL, HE-A, and SM: formal analysis. AAM, IJ, GT, HK, and DK: investigation. AAM, IJ, GT, HK, DK, and HE-A: resources. AAM, JL, HE-A, HT, SM, and HN: data curation. AAM: writing—original draft preparation. AAM, HE-A, JL, SM, HT, and HN: writing—review and editing, visualization, and supervision. All authors contributed to the article and approved the submitted version.

## Funding

This research was funded by the German Federal Ministry of Education and Research (BMBF) and the German Federal Ministry for Economic Affairs and Energy, within the framework of joint project “Adaptable decentralized diagnostics for veterinary and human medicine (ADA)”; Subproject: Adaptable diagnostics for prudent antibiosis of mastitis in livestock (ADA-M) (Funding No.: 13GW0456D: BMBF).

## Conflict of interest

The authors declare that the research was conducted in the absence of any commercial or financial relationships that could be construed as a potential conflict of interest.

## Publisher’s note

All claims expressed in this article are solely those of the authors and do not necessarily represent those of their affiliated organizations, or those of the publisher, the editors and the reviewers. Any product that may be evaluated in this article, or claim that may be made by its manufacturer, is not guaranteed or endorsed by the publisher.

## Footnotes

^1^ https://gitlab.com/FLI_Bioinfo/WGSBAC

^2^ https://github.com/tseemann/shovill

^3^ https://github.com/tseemann/abricate

^4^ https://pubmlst.org/organisms/staphylococcus-aureus

^5^ https://github.com/tseemann/snippy

^6^ https://itol.embl.de/login.cgi
